# Arterial access-site complications after use of a vascular closure device related to puncture height

**DOI:** 10.1186/s12872-017-0484-7

**Published:** 2017-02-16

**Authors:** Benjamin Sartorius, Michael Behnes, Melike Ünsal, Ursula Hoffmann, Siegfried Lang, Kambis Mashayekhi, Martin Borggrefe, Ibrahim Akin

**Affiliations:** 1 First Department of Medicine, University Medical Centre Mannheim (UMM), Faculty of Medicine Mannheim, University of Heidelberg, Theodor-Kutzer-Ufer 1-3, 68167 Mannheim, Germany; 2 Department of Cardiology and Angiology II, University Heart Center Freiburg - Bad Krozingen, Bad Krozingen, Germany

**Keywords:** Bleeding, Vascular closure device, Femoral artery

## Abstract

**Background:**

To analyze differences of access-site complications related to the height of femoral arterial puncture and the use of a vascular closure device (VCD) following percutaneous coronary intervention (PCI).

**Methods:**

A subgroup of the FERARI study being treated by femoral arterial access and valuable inguinal angiography before implantation of a VCD were included. Inguinal angiographies were systematically reviewed by two independent cardiologists to determine the correct height of femoral arterial puncture. Bleeding complications were documented within 30 days after PCI and were categorized according to BARC, TIMI, GUSTO and FERARI classifications.

**Results:**

Femoral access point imaging was available in 95 patients compared to 105 patients without. The common femoral artery (CFA) was the most accessed artery in 41%, followed by the femoral arterial bifurcation (39%) and lower access sites distally from the femoral arterial bifurcation (low puncture: 20%). No differences were observed regarding indication of PCI, procedural data and anticoagulation therapies in relation to the heights of femoral arterial access (*p* > 0.05). Despite using VCD, arterial puncture at the CFA resulted in numerically highest numbers of overall bleedings (62%) compared to femoral arterial bifurcation (41%) (*p* = 0.059). 58% of bleedings occurred after arterial puncture below the femoral bifurcation (low puncture). Though no significant differences of bleedings regarding classifications of BARC, GUSTO, TIMI and FERARI as well as other vascular endpoints were observed regarding puncture height.

**Conclusions:**

The present analysis demonstrates no significant differences of bleeding complications in relation to the height of femoral arterial puncture and subsequent use of a VCD.

## Background

Bleedings are a common complication of percutaneous coronary interventions (PCI) both affecting the arterial access site and occurring in terms of general outcome. Bleedings do not only affect patients’ satisfaction, but do also increase mortality [1]. With specific regard to femoral arterial access-site bleedings, different strategies have been evaluated in order to reduce them. Besides manual compression and application of pressure bandages around the hips, so called vascular closure devices (VCD) were developed in the early 1990’s [2]. VCD were shown to reduce access-site bleedings and to reduce post interventional time to hemostasis [3, 4]. In contrast non-access site bleedings are supposed to result in a higher mortality risk compared to bleedings from primary access site [5]. Vavalle et al. showed in-hospital bleedings in acute coronary syndrome (ACS) patients to be associated with an increased risk of death, whereas mild access-site bleedings were not [6].

VCD have entered the medial practice in modern cardiac catheterization laboratories [3, 7]. Additionally this treatment has proven advantageous on the patients’ overall satisfaction and mobilization [8]. Different characteristics were shown to predict arterial access-site complications after PCI, such as advanced age, female sex, bleeding predispositions or renal failure [9, 10]. However, there is no evidence currently available on how the height of femoral arterial puncture alters the occurrence of PCI-related bleedings.

Therefore, this sub-study of the FERARI trial [11, 12] aims to evaluate the differences of PCI-related bleedings depending on the height of femoral arterial puncture in combination with the treatment of a VCD.

## Methods

### Study patients and data collection

**Fig. 1:**
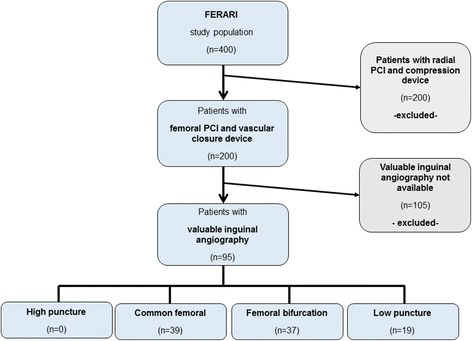
Flow chart illustrating the selection of patients for the present sub-analysis of the FERARI study

The present study is based on a subset of patients from the “Femoral Closure versus Radial Compression Devices Related to Percutaneous Coronary Interventions (FERARI)” study. The FERARI study is a single-center prospective, nonrandomized observational study being performed at the First Department of Medicine, University Medical Centre Mannheim (UMM) in Mannheim, Germany (clinicaltrials.gov identifier: NCT02455661). The study was approved by the medical ethics commission II of the Faculty of Medicine Mannheim, University of Heidelberg, Germany. Written informed consent is obtained from all participating patients or their legal representatives [11]. Briefly, those patients with a valuable inguinal angiography before successful implantation of the AngioSealTM femoral VCD (Angio-Seal™; St. Jude Medical, Inc., St Paul, USA) following PCI were included. Patients with radial PCI and patients with a femoral PCI without any or a valuable inguinal angiography were excluded (Fig. 1). Performing femoral angiography belonged to the operators’ discretion and was performed to verify the location of puncture above or below the epigastric artery, to detect severe calcification of the puncture site or to alleviate difficult femoral or aortic passing.

Standardized angiography was performed from left anterior oblique (LAO) 30° and right anterior oblique (RAO) 30° view. A valuable inguinal angiography was defined, when the entire inguinal anatomy of the iliac and femoral arteries was documented, including the femoral bifurcation. All angiographies were systematically reviewed on the “OsiriX Imaging Software” (Pixmeo SARL, Switzerland) by two independent cardiologists in order to determine the correct height of femoral arterial puncture.

Femoral arterial puncture was performed by standardized Seldinger technique in all patients using a conventional stainless steel puncture needle (SFM Medical Devices©, Wächtersbach, Germany) (i.e. no micropuncture needle). Ultrasound guiding during arterial puncture or concomitant ipsilateral venous or arterial access was excluded. Number of needle passes was not documented, but was restricted to a maximum number of 5 in cases of difficult anatomy or calcification. The allowed French size in the FERARI study was 5–6 French. Only four different experienced interventional cardiologists performed PCIs in this study with a minimum volume of 300 PCI cases per year and an equal distribution of experiences with both the radial and femoral approach of each operator. Operators had already implanted at least 100 AngioSeal© femoral closure devices per year. All operators were equally experienced in both radial and femoral accessed interventions [11].

### Definition of height of femoral arterial puncture based on anatomical description

The procedural X-ray contrast angiography of the femoral arteries was reviewed systematically and the access point was described anatomically. Arterial parts were classified into the external iliac artery (EIA), common femoral artery (CFA), femoral bifurcation, profound femoral artery (PFA) and superficial femoral artery (SFA). Based on these anatomic landmarks 4 different puncture heights were defined: high puncture at the EIA and above the inguinal ligament, femoral puncture at the CFA, femoral bifurcation and low puncture below the femoral bifurcation either at the PFA or SFA.

The border between EIA and CFA was drawn at the superficial epigastric artery as an indicator for the localization of the inguinal ligament. The femoral bifurcation was described as the area of 1 cm into each proximal and distal vessel branches. The PFA and SFA were distinguished by the typical lateral projection of the prominently visual course of the PFA and its side branches, namely the lateral circumflex femoral artery and the medial circumflex femoral artery.

### Post interventional procedure

After successful PCI the VCD was implanted. Afterwards a circumferential pressure bandage is added to the puncture site to further support the prevention of bleedings. Patients were advised to remain in lying position (i.e. bed-rest) for 6 h. Pressure bandages were removed after a total of 12 h, unless access site bleedings occurred. Patients were not subject to early ambulation. All PCI patients remained hospitalized for at least 24 h, early ambulation was not allowed after PCI.

### Outcome definition and follow-up

All patients were followed up during hospital stay and until 30-days after the index procedure directly and by standardized telephone visits. The primary outcome was defined as overall, access-site and non-access-site bleedings occurring within 30-days of follow-up. Overall bleedings were defined according to established bleeding classifications, i.e. “Bleeding Academic Research Consortium” (BARC) score, “The Thrombolysis in Myocardial infarction” (TIMI) score and the “Global Use of Strategies to Open Occluded Arteries” (GUSTO) score [6, 13, 14]. Access site bleedings were defined as hematomas, active bleedings, dissections, pseudoaneurysms, arteriovenous fistula, retroperitoneal hematoma and non-access-site bleeding. Access site bleedings were classified according to the study-specific FERARI classification, as described previously: [11] small (<5 cm), intermediate (5–15 cm), large (>15 cm) hematomas and complicated access-site bleedings.

### Statistics

Statistical analyses were performed with SPSS Statistics (IBM, Armonk, NY) and GraphPad Prism (GraphPad Software, Inc., La Jolla, CA). Data are presented as medians with interquartile ranges (25th and 75th percentiles) or as total numbers with group-related percentages. Normal distribution of data was tested with the Kolmogorov-Smirnov test. In case of normal distribution, the *t*-test was applied to compare scaled data. Scaled variables not normally distributed were compared using the Mann–Whitney *U* test. Categorical variables were compared using the chi-squared test, in case of low event rates the Fisher’s exact test was used. Risk factors for access site bleedings, which were shown to differ significantly between the two study groups, were adjusted using uni- and multivariate linear regression analyses for the predefined study outcomes representing the dependent variable. Level of significance was set at *p* < 0.05 (two-tailed), a statistical trend was defined at *p* < 0.1 (two-tailed).

## Results

### Study population

As illustrated in Fig. 1, of the initial study cohort of 400 patients, final number of 95 patients revealed valuable inguinal angiography as well as successful femoral PCI with a femoral VCD and were included for the present sub-analysis.

**Fig. 2:**
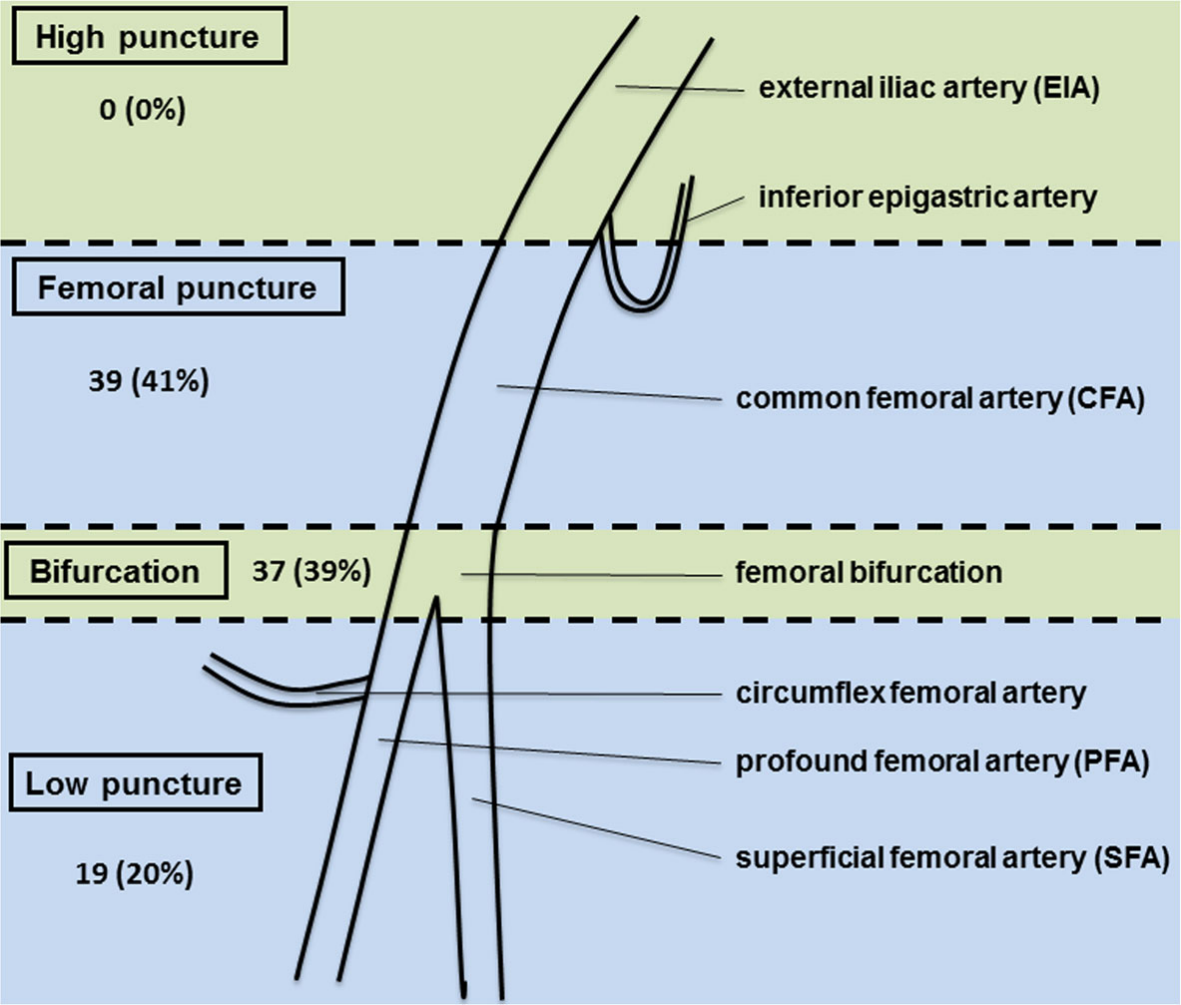
Topographic scheme demonstrating the different heights of femoral arterial puncture as well as the distribution of puncture rates being evaluated from inguinal angiographies following PCI. Puncture rates are presented as n (%)

Figure 2, illustrates the distribution of the different arterial puncture sites. CFA was the most punctured arterial site (41%), followed by the femoral bifurcation (39%) and low puncture distally to the femoral bifurcation either at the PFA or SFA (20%). High punctures above the inguinal ligament were not performed within the present cohort.

Patient demographics were equally distributed between groups (Table 1), with a mean age of 67 to 71 years. Patients were often male, being mostly present in all subgroups (80% in common femoral, 64% in femoral bifurcation), despite in patients of the low puncture group (37%) (*p* = 0.006). Body mass index (BMI) was higher in femoral punctured patients compared to patients in the low puncture groups (*p* = 0.038). No further significant differences were found regarding cardiovascular risk factors between subgroups (Table 1). Accordingly the indications for PCI as well as mean procedural length or implanted stent lengths did not differ significantly (Table 1). The hospital stay time averaged at 9 days. During the 30 day follow-up period 11% of patients were rehospitalized, 4% received additional coronary angiography, while 3% received additional stent implantation. One stent thrombosis was documented, which resulted in the only death (1%).

**Table 1:** Baseline characteristics of the study population

	All patients (*n* = 95)	Common femoral artery (*n* = 39)	Femoral bifurcation (*n* = 37)	Low puncture (*n* = 19)	*p* value
Age, mean ± SD	69.3 ± 10.5	67 ± 10.6	70.7 ± 9.7	71.4 ± 11.4	0.142
BMI, mean ± SD	28.3 ± 5.6	26.4 ± 3.7	29.5 ± 7.2	29.6 ± 4.9	0.038
Male, n (%)	61 (64)	31 (80)	23 (64)	7 (37)	0.006
Cardiovascular risk factors, n (%)					
Arterial hypertension	71 (75)	26 (67)	31 (86)	14 (74)	0.228
Diabetes	34 (35)	9 (23)	16 (44)	9 (47)	0.089
Smoking	16 (17)	10 (26)	3 (8)	3 (16)	0.118
Cardiac family history	12 (13)	3 (8)	6 (16)	3 (16)	0.484
Prior medical history, n (%)					
Congestive heart failure	12 (13)	5 (13)	5 (14)	2 (11)	0.922
Coronary artery disease	46 (48)	16 (41)	20 (54)	10 (53)	0.482
Peripheral artery disease	6 (6)	3 (8)	2 (5)	1 (5)	1.000
Chronic kidney disease	21 (22)	5 (13)	10 (27)	6 (32)	0.159
Indication for PCI, n (%)					
STEMI	6 (6)	2 (5)	2 (5)	2 (11)	0.741
NSTEMI	27 (28)	12 (31)	11 (30)	4 (21)	0.726
Unstable AP	19 (20)	9 (24)	6 (16)	4 (21)	0.748
Stable AP	19 (20)	6 (15)	8 (21)	5 (26)	0.592
Other	24 (25)	10 (26)	10 (27)	4 (21)	0.887
Procedural data					
Average Time	47	52	51	50	
Drug eluting stents, n (%)	85 (90)	35 (90)	33 (89)	17 (90)	1.000
Bare metal stents, n (%)	4 (4)	1 (3)	2 (5)	1 (5)	0.836
Total stent length, mean ± SD	42.4 ± 29.7	39.5 ± 24.8	42.3 ± 31.7	48.3 ± 30.6	0.561

Table 2 summarizes the different anticoagulant therapies being administered for all spectated bleedings. Neither pre-, peri- nor post-interventional combinations of anticoagulant therapies revealed any significant differences related to the different groups of arterial puncture height.

**Table 2:** Anticoagulation of patients with recorded bleedings during PCI

	All patients (*n* = 50)	Common femoral artery (*n* = 24)	Femoral bifurcation (*n* = 15)	Low puncture (*n* = 11)	*p* value
Pre-PCI, n (%)					
ASS	27 (54)	11 (46)	10 (67)	6 (55)	0.447
Clopiogrel	9 (18)	5 (21)	1 (7)	3 (27)	0.346
Ticagrelor	1 (2)	0 (0)	1 (7)	0 (0)	0.520
Prasugrel	5 (10)	1 (4)	3 (20)	1 (9)	0.284
LMH	2 (4)	0 (0)	1 (6)	1 (9)	0.265
NOAC/OAC	8 (16)	4 (17)	3 (20)	1 (9)	0.886
None	17 (34)	11 (46)	2 (13)	4 (36)	0.104
INR, mean ± SD	1.07 ± 0.23	1.09 ± 0.26	1 ± 0.08	1.16 ± 0.33	0.066
Peri-PCI, n (%)					
Heparin	50 (100)	24 (100)	15 (100)	11 (100)	1
GP IIB/IIIA inhibitor	2 (4)	1 (4)	0 (0)	1 (9)	0.481
Bivalirudin	1 (2)	0 (0)	1 (7)	0 (0)	0.520
Post-PCI, n (%)					
ASS + Clopidogrel	33 (66)	14 (58)	10 (67)	9 (82)	0.395
ASS + Ticagrelor	4 (8)	1 (4)	2 (13)	1 (9)	0.545
ASS + Presugrel	6 (12)	5 (21)	1 (7)	0 (0)	0.279
ASS + NOAC/OAC	0 (0)	0 (0)	0 (0)	0 (0)	-
Thienopyridine + NOAC/OAC	3 (6)	1 (4)	2 (13)	0 (0)	0.432
Triple Therapy	4 (8)	3 (13)	0 (0)	1 (9)	0.424

*LMH* low molecular heparin, *NOAC/OAC* Warfarin or one of the new oral anticoagulants (Rivaroxaban, Dabigatran, Apixaban); Triple therapy: ASS + Clopidogrel + NOAC/OAC

### Bleedings related to the height of femoral arterial puncture

Overall bleedings occurred in more than half of all patients (53%) with prior femoral angiography, which was not significantly different compared to patients without femoral angiography (n = 105, overall bleeding rate 58%) (*p* = 0.437) (Table 3). Most patients received 6 F introducer sheaths (femoral angiography: 85% versus 15%; no femoral angiography: 95% versus 5%). No differences of bleeding rates were found depending on the choice of sheath size or presence of angiography (*p* > 0.05).

**Table 3:** Bleeding rates depending on the presence of femoral angiography in PCI patients with a femoral VCD

	Angiography (*n* = 95)	No angiography (*n* = 105)	*p* value
Overall bleedings, n (%)	50 (53)	61 (58)	0.437
Related to 5 F, ratio (%)	6/14 (43)	7/15 (47)	0.837
Related to 6 F, ratio (%)	44/81 (54)	54/90 (60)	0.453
BARC, n (%)			
0	45 (48)	44 (42)	0.437
1	38 (40)	50 (48)	0.278
2	10 (11)	10 (10)	0.813
3A	0 (0)	1 (1)	1
3B	2 (2)	0 (0)	0.224
3C	0 (0)	0 (0)	-
4	0 (0)	0 (0)	-
5	0 (0)	0 (0)	-
TIMI, n (%)			
0	45 (47)	44 (42)	0.437
1	50 (53)	60 (57)	0.522
2	0 (0)	1 (1)	1
3	0 (0)	0 (0)	-
GUSTO, n (%)			
0	45 (47)	44 (42)	0.437
1	49 (52)	60 (57)	0
2	0 (0)	1 (1)	1
3	1 (1)	0 (0)	0.475
FERARI, n (%)			
0	45 (47)	44 (42)	0.437
1	25 (26)	27 (26)	0.923
2	13 (14)	10 (10)	0.681
3	9 (10)	11 (10)	0.813
4	3 (3)	3 (3)	1

With regard to BARC, TIMI and GUSTO bleeding classifications 52.6% patients received a score of 1 or higher (Tables 3 and 4). Moderate bleedings (GUSTO 2) did not develop, whereas a severe bleeding (GUSTO 3) was only observed once (1.1%) in the common femoral subgroup. Including all primary access site bleedings, 61.5% femoral punctured patients showed bleedings (BARC, TIMI, GUSTO score ≥ 1). In the femoral bifurcation group bleedings were spectated in 41% patients, low puncture showed 58% bleedings (Tables 3 and 4). Mostly observed bleedings consisted of local hematomas (FERARI class 1–3) in 49.5% patients. Complicated access-site bleedings consisted of post-interventional open bleedings (2.1%), AV-dissection (2.1%), aneurysm spurium (1.1%).

**Table 4:** Bleedings according to heights of femoral arterial punctures

	Common femoral artery (*n* = 39)	Femoral bifurcation (*n* = 37)	Low puncture (*n* = 19)	*p* value
Overall bleedings, n (%)	24 (62)	15 (41)	11 (58)	0.163
Related to 5 F, ratio (%)	2/3 (67)	2/8 (25)	3/3 (100)	0.143
Related to 6 F, ratio (%)	22/36 (61)	13/29 (45)	8/16 (50)	0.409
BARC, n (%)				
0	15 (38)	22 (60)	8 (42)	0.163
1	16 (41)	13 (35)	9 (47)	0.667
2	7 (18)	2 (5)	1 (5)	0.191
3A	0 (0)	0 (0)	0 (0)	-
3B	1 (3)	0 (0)	1 (5)	0.677
3C	0 (0)	0 (0)	0 (0)	-
4	0 (0)	0 (0)	0 (0)	-
5	0 (0)	0 (0)	0 (0)	-
TIMI, n (%)				
0	15 (38)	22 (59)	8 (42)	0.163
1	24 (62)	15 (41)	11 (58)	0.163
2	0 (0)	0 (0)	0 (0)	-
3	0 (0)	0 (0)	0 (0)	-
GUSTO, n (%)	2 (5)	2 (5)	2 (11)	0.741
0	15 (39)	22 (60)	8 (42)	0.163
1	23 (59)	15 (41)	11 (58)	0.228
2	0 (0)	0 (0)	0 (0)	-
3	1 (3)	0 (0)	0 (0)	1.000
FERARI, n (%)				
0	15 (38)	22 (60)	8 (42)	0.163
1	12 (31)	8 (22)	5 (26)	0.663
2	6 (15)	6 (16)	1 (5)	0.487
3	5 (13)	1 (3)	3 (16)	0.143
4	1 (3)	0 (0)	2 (11)	0.101

## Discussion

The present subanalysis of the FERARI study evaluates differences of PCI-related bleedings depending on the height of femoral arterial puncture in combination with the treatment of a femoral VCD. It was demonstrated that the common femoral artery and femoral bifurcation were the most punctured access sites in the present cohort possibly revealing easiest anatomical accessibility. Arterial punctures below the femoral bifurcation was less performed. Accordingly, arterial puncture at the bifurcation revealed a numerically lowest amount of bleedings (41%) compared to the CFA (62%) and lower punctures (58%), without reaching statistical cut-off.

In contrast, lower arterial punctures could not be shown to be associated with a worse outcome. It might me speculated whether a lower arterial puncture might be a valid alternative puncture site without increasingly risking bleedings.

No significant differences of baseline characteristics, indications of PCI and anticoagulation therapies were seen according to the described femoral arterial puncture sites, with the exception of a significantly higher number of female patients in the lower puncture group. This might indicate, that female patients are more likely to be punctured distally of the femoral bifurcation than male patients. Comparing female and male bleeding probabilities, no significance was recorded in the present study, although former studies have shown female gender having an increased bleeding risk [9]. Therefore the high number of bleedings in the lower puncture group might partially be a result of the high percentage of female patients (63%). The BMI differences might indicate that increased weight moves the operator to perform arterial puncture more distally. Additionally overweight and obese patients have lower bleeding rates [15].

The extend of documented bleedings was rarely of severe nature. Whether severe bleedings are associated similarly by different arterial puncture sites therefore needs to be investigated by large scale studies. The evaluation of anticoagulation therapies did not show any significant differences in each evaluated subgroup, making an uneven influence on the observed bleedings unlikely.

### Study limitations

Patients were included retrospectively in the FERARI study after successful PCI and either VCD implantation or radial compression bracelet usage and therefore preselected and non-randomized. Only a single type of VCD was used in this study. Unsuccessful VCD implantations were not included into the FERARI study and could therefore not be analysed. Follow up period was limited to 30 days, which does not give any information on long term effects. Finally, bleeding events were only described clinically, without a standardized imaging technique such as duplex sonography.

## Conclusions

In conclusion, the present study suggests, that bleeding risks associated with different punctured femoral arterial sites does not differ significantly. Therefore, neither CFA, femoral arterial bifurcation, nor profound and superficial femoral artery might have favoured the occurrence of bleeding. Weather the statistical trend being observed for a less bleeding rate at the femoral bifurcation is just statistical variance or due to a low study population needs to be investigated within a larger prospective studies.

## Abbreviations

BARC: Bleeding Academic Research Consortium
CFA: Common femoral artery
FERARI: Femoral Closure versus Radial Compression Devices Related to Percutaneous Coronary Interventions
GUSTO: The Global Use of Strategies to Open Occluded Arteries
NOAC: New oral anticoagulants
PFA: Profound femoral artery
SFA: Superficial femoral artery
TIMI: Thrombolysis in Myocardial Infarction
